# Integrative Transcriptomics and Proteomics Analysis Reveals *THRSP*’s Role in Lipid Metabolism

**DOI:** 10.3390/genes15121562

**Published:** 2024-11-30

**Authors:** Yujie Li, Ke Xu, Ao Zhou, Zhong Xu, Junjing Wu, Xianwen Peng, Shuqi Mei, Hongbo Chen

**Affiliations:** 1 Laboratory of Genetic Breeding, Reproduction and Precision Livestock Farming, School of Animal Science and Nutritional Engineering, Wuhan Polytechnic University, Wuhan 430023, China; liyujie13653814659@163.com (Y.L.); 22113033@whpu.edu.cn (K.X.); aoqiu@whpu.edu.cn (A.Z.); 2 Hubei Provincial Center of Technology Innovation for Domestic Animal Breeding, Wuhan Polytechnic University, Wuhan 430023, China; 3 Key Laboratory of Animal Embryo Engineering and Molecular Breeding of Hubei Province, Wuhan 430064, China; xz8907@163.com (Z.X.); jeanne1106@126.com (J.W.); pxwpal@163.com (X.P.)

**Keywords:** *THRSP*, lipid metabolism, endoplasmic reticulum stress, lipid synthesis

## Abstract

**Background/Objectives:** Abnormalities in lipid metabolism and endoplasmic reticulum (ER) stress are strongly associated with the development of a multitude of pathological conditions, including nonalcoholic fatty liver disease (NAFLD), diabetes mellitus, and obesity. Previous studies have indicated a potential connection between thyroid hormone responsive (*THRSP)* and lipid metabolism and that ER stress may participate in the synthesis of key regulators of adipogenesis. However, the specific mechanisms remain to be investigated. **Methods:** In this study, we explored the roles of *THRSP* in lipid metabolism by interfering with *THRSP* gene expression in mouse mesenchymal stem cells, comparing the effects on adipogenesis between control and interfered groups, and by combining transcriptomic and proteomic analysis. **Results:** Our results showed that the number of lipid droplets was significantly reduced after interfering with *THRSP*, and the expression levels of key regulators of adipogenesis, such as *LPL*, *FABP4*, *PLIN1*, and *CIDEC*, were significantly downregulated. Both transcriptomic and proteomic results showed that the differential genes (proteins) were enriched in the processes of lipolytic regulation, ER stress, cholesterol metabolism, sphingolipid metabolism, PPAR signaling pathway, and glycerophospholipid metabolism. The ER stress marker gene, *ATF6*, was the most significantly downregulated transcription factor. In addition, RT-qPCR validation indicated that the expression levels of PPAR signaling pathway gene *SCD1*; key genes of lipid droplet generation including *LIPE*, *DGAT1*, and *AGPAT2*; and ER stress marker gene *ATF6* were significantly downregulated. **Conclusions:** These suggest that *THRSP* is involved in regulating ER stress and the PPAR signaling pathway, which is closely related to lipid synthesis and metabolism. Interfering with the expression of *THRSP* may be helpful in ameliorating the occurrence of diseases related to abnormalities in lipid metabolism.

## 1. Introduction

Metabolic diseases are disorders that affect the body’s normal metabolic processes and commonly include diabetes, NAFLD, and atherosclerosis [1,2,3]. Of these, diabetes is often accompanied by insulin resistance and hyperglycemia, which are closely associated with abnormal lipid metabolism [4]. For atherosclerosis, altered lipid metabolism is considered a key factor [5]. Fat deposition is an important feature of metabolic diseases, especially NAFLD [6,7]. Lipid metabolism in the liver is controlled by factors, including the synthesis of fatty acids and the process of β-oxidation. When these metabolisms are dysregulated, lipid accumulation in the liver can occur, triggering inflammation and fibrosis [8,9,10].

ER, the main cellular organelle of lipid synthesis, directly affects the balance of lipid metabolism with its normal or abnormal function [11]. ER dysfunction triggers ER stress, which in turn activates inflammatory signaling pathways and leads to metabolic diseases [12,13,14]. It has been demonstrated that ER stress exerts a pivotal role in the synthesis of lipids. For example, the inhibition of ER stress marker gene *ATF6* expression in mesenchymal stromal C3H10T1/2 (C3H10) cells leads to the downregulation of *PPARγ* in the PPAR signaling pathway and reduces lipogenesis; overexpression of *ATF6* in mouse embryonic fibroblasts (MEFs) upregulates the expression of *ACACB* and *FASN*, which are regulators of lipid metabolism. Therefore, the study of factors affecting lipid metabolism is important for the prevention and management of illnesses associated with abnormal lipid metabolism.

The *THRSP* gene is a type of thyroid hormone-induced liver protein that can form different dimers with *S14R* (*MID1*, *MIG12*) and is involved in regulating lipid biosynthesis. *THRSP* and *S14R* can bind to acetyl-CoA carboxyl-ase (ACC), the rate-limiting enzyme of fatty acid synthesis, to enhance the enzymatic activity of ACC, and then upregulate hepatic fatty acid synthesis and triglyceride content [15]. *THRSP* is also involved in glycolysis and the glucose production pathway. It is closely associated with liver fat production. According to previous literature reports, *THRSP* has been found to participate in the pathogenesis of NAFLD [16]. Overexpression of *THRSP* in the liver has been shown to increase the accumulation of triglycerides in the liver of mice and enhance fat generation, and its silence can weaken liver steatosis [17,18]. The role of *THRSP* in fatty acid oxidation has also received attention. It has been found that the fatty acid oxidation of radiooleic acid as a substrate was significantly reduced in *THRSP*-silenced cells, indicating that *THRSP* may serve as a facilitative factor in fatty acid metabolism [19,20]. Some studies have identified *THRSP* as a key gene related to fat deposition, and miR-195 can affect fat deposition in buffalo by inhibiting *THRSP* [21]. In addition, *THRSP* has been closely associated with fat deposition in studies of meat shape in both livestock and poultry, such as goats, pigs, and broilers [22,23,24,25]. In our preceding study, we discovered that the expression level and ATAC-seq peak of *THRSP* were markedly elevated in a group of Xidu black pigs with high intramuscular fat (IMF) content [26], suggesting *THRSP* is associated with adipogenic mechanisms.

However, the effects of *THRSP* on adipogenesis in mouse mesenchymal stem cell line have not been described. Thus, to better understand the mechanism by which the *THRSP* gene affects lipid synthesis in adipose progenitor cells, we investigated the effect of interfering with *THRSP* gene expression on the expression of lipid synthesis-related genes, and we explored the specific mechanism by combining transcriptome sequencing and proteome technologies. The results provide a theoretical basis for *THRSP* regulation in understanding lipid metabolism in animals.

## 2. Materials and Methods

### 2.1. Cell Culture, Transfection and Differentiation

C3H10 cells are mouse mesenchymal stem cells line preserved in our laboratory. The cells were cultured on Dulbecco’s Modified Eagle Medium Nutrient Mixture Fly12 (DMEM-F12, gibco) containing 10% Fetal Bovine Serum (FBS) premium Plus (gibco, New York, NY, USA) and 1% cell culture triple antibody (PSS) 100× (HANBIO). All cells were cultured in a 5% CO_2_ incubator at 37 °C.

The cells were inoculated in 6-well/24-well plates. When the cell density reached 80%, the cells were transfected according to the instructions for Lipofectamine^®^ RNAiMAX Reageni (Thermo Fisher Scientific, Beijing, China) reagent. siRNA was diluted to 20 μM. *THRSP*-siRNA was synthesized by Suzhou Jima Genome Co., Ltd (Suzhou, China). (sense (5-3′): GAGAACGACGCUGCUGAAATT, antisense (5′-3′): UUUCAGCAGCGUCGUUCUCTT).

After 24 h of transfection, the C3H10 cell culture medium was replaced by classical MDI+ indomethacin induction medium (DMEM-F12 differentiation induction medium containing 0.1 mmol/L 3-isobutyl-1-methylxanthine (IBMX), 1 μmol/L dexamethasone, 10 mg/L insulin, 10% fetal bovine serum, and 0.1 mmol/L indomethacin) for 48 h [27,28].

### 2.2. Quantitative Real-Time PCR (RT-qPCR)

Total cell RNA extraction was performed using the E.Z.N.A.^®^ HP Total RNA Kit instructions (Omega Bio-tek, Norcross, GA, USA). The cDNA was prepared using ABScript III RT Master Mix for qPCR with gDNA Remover (ABclonal, RK20429, Wuhan, China). The prepared cDNA was used to detect gene expression by quantitative real-time PCR (RT-qPCR) using 2X Universal SYBR Green Fast qPCR Mix (ABclonal, RK21203) reagent, and gene expression was detected by RT-qPCR. The sequence of primers used is shown in Table S1. Finally, the 2^−ΔΔCt^ method was used for data analysis and one-way analysis of variance. The *p*-values were obtained by calculating the *t*-test (Student’s *t* test) through GraphPad Prism 9.5.0. *p* < 0.05 was considered significant difference; *p* < 0.01 was considered extremely significant difference.

### 2.3. BODIPY and Oil-Red-O Staining

C3H10 cells were stained with BODIPY (Thermo Fisher, Shanghai, China) and Oil-RED-O (Improved Oil Red O staining kit, Biyun Tian, Shanghai, China) 8 days after induction, in accordance with the instructions provided in the kit.

### 2.4. Effects of Interference with THRSP on Lipid Formation Analysed by Transcriptomic Techniques

#### 2.4.1. Quality Control of Transcriptome Sequencing Results

The raw data obtained by sequencing contained a small number of reads with sequencing adapters or lower sequencing quality. In order to ensure the quality and reliability of data analysis, the raw data were filtered using fastp (v0.12.4) [29] software as follows:

i. Removal of reads with adapters;
ii. Removal of reads containing N (N means the base information is unknown);
iii. Removal of low-quality reads (reads where more than 50% of bases had a Qphred score of less than 20).

#### 2.4.2. Reference Genome Alignment

The clean reads were rapidly and accurately mapped to the reference genome using HISAT2 [30] software (Official Manual of HISAT2 (v2.2.1) Software) to attain information on the positioning of the reads on the reference genome (Mus musculus GRCm39 release-109, downloaded from https://ftp.ensembl.org/pub/release-109/fasta/mus_musculus/ (accessed on 20 May 2024)).

#### 2.4.3. Library Quality Assessment

The randomness of mRNA fragmentation was investigated by means of a simulation based on the positional distribution of mapped reads on each mRNA transcript.

#### 2.4.4. Quantitative Gene Expression Analysis

The feature count tool in the subread software was used to calculate the expression level of the gene based on the number (count) of sequencing reads compared to each transcript. The number of reads covering the start to the end range of each gene was counted based on the position information of the gene ratio on the reference genome. The number of mapped reads in the samples and the transcript length were normalized. FPKM (fragments per kilobase of transcript per million fragments mapped) was used to normalize the raw read count value as a measure of transcript or gene expression level.

#### 2.4.5. Gene Differential Expression Analysis

An analysis of variance was conducted using DESeq2 (v1.2.10) [31] software. The screening criteria employed were a fold change > 2 and a *p*_adj_ < 0.05. The multiplicity of difference (fold change) represents the ratio of expression between two samples (groups). For ease of comparison, the multiplicity of difference was taken as a logarithmic value and expressed as log_2_FC. GO and KEGG enrichment analyses were conducted using the hypergeometric test, respectively, with the aid of Cluster Profiler software (v4.14.3).

### 2.5. Quantitative Proteomics Analysis

#### 2.5.1. Sample Protein Extraction

The protein sample was taken from the refrigerated (−80 °C) storage and thawed on ice, and the cell lysate (containing 1% protease inhibitor) was then added. Following ultrasonic crushing, the supernatant was subjected to centrifugation at 15,000× *g* for 10 min, the impurities were removed, and the supernatant was transferred to a new centrifuge tube. The protein concentration of supernatant protein was determined by BCA.

#### 2.5.2. Enzyme Digestion

Precipitation enzyme digestion: Based on the protein concentration determination value, an equal amount of protein was taken from each sample, and the volume was made up with lysate to the same volume. The appropriate volume of TCA was then slowly added to make the final concentration 20%. The mixture was fully vortex-mixed and then left to precipitate at 4 °C for a period of 2 h. Centrifugation was carried out at 4 °C and 15,000× *g* for 5 min. The protein precipitate was then collected and washed with −20 °C pre-cooled acetone 3 times. Once the acetone had evaporated and the precipitate had been dried, it was suspended in 100 mM TEAB, reconstituted by sonication in a water bath, and trypsin was added at a ratio of 1:50 (protease: protein, m/m). The mixture was then digested at 37 °C overnight. The final concentration of 5 mM dithiothreitol (DTT) was added at 56 °C for a reduction period of 30 min. Subsequently, the final concentration of 15 mM iodoacetamide (IAA) was added at room temperature for a reaction period of 15 min, with the utmost care taken to avoid exposure to light. Once the reaction had been completed, the appropriate volume of 10% TFA was added to the sample in order to achieve an acidic pH value of 2–3, with Strata X (Phenomenex, Fujian, China) demineralization following real-air freezing. The peptides were fully re-solubilized in water and quantified using the Pierce™ Quantitative Peptide Assays & Standards kit (Thermo Scientific, Shanghai, China).

#### 2.5.3. LC-MS/MS Analysis

The analysis utilized the nanoflow rate Vanquish Neo system (Thermo Fisher Scientific) for chromatographic separation, and samples post-high-efficiency liquid chromatography separation were subjected to data independent acquisition (DIA) mass spectrometry using an Astral high-resolution mass spectrometer (Thermo Fisher Scientific). The detection mode was positive ionisation, with a precursor ion scan range of 380–980 m/z, and a primary mass spectrometry resolution of 240,000 at 200 m/z. The normalized AGC target was set at 500%, with a maximum IT of 5 ms. MS2 employed the DIA data acquisition mode, with 299 scan windows set, an isolation window of 2 m/z, HCD collision energy set at 25 ev, normalized AGC target at 500%, and a maximum IT of 3 ms.

#### 2.5.4. Database Search

The DIA data were processed using DIA-NN (v1.9) software. The software parameters were set as follows: the enzyme used was trypsin, with a maximum missed cleavage site set to 1. The fixed modification was carbamidomethyl (C), and dynamic modifications were set to oxidation (M) and acetyl (protein N-term). Proteins identified through database retrieval had to pass the set filtering parameter of FDR < 1%.

#### 2.5.5. Bioinformatics Analysis

Quality control analyses were performed at the peptide and protein level, utilizing the findings of database searches as a reference. The functional annotations of identified proteins were drawn from a number of sources, including GO, KEGG, KOG (Clusters of orthologous groups for eukaryotic complete genomes), protein domain, and STRING database annotations. The quantitative analysis of proteins comprised the following elements: quantitative distribution statistics and reproducibility analysis. The latter was assessed using three statistical analyses. The principal component analysis (PCA), Pearson correlation coefficient (PCC) and relative standard deviation (RSD) were employed in the analysis. The differentially expressed proteins (DEPs) screening process entailed a series of analytical procedures, including a significance test for differences, a statistical analysis of the number of differences, the construction of differential protein volcano plots, and the generation of expression heat maps. The differential protein functional classification statistical analysis comprised GO, KEGG pathway, subcellular localization classification, and KOG classification statistics. Based on the classification statistics, Fisher’s exact test was employed for the purpose of calculating the significantly enriched biological functions or pathways of differential proteins. A protein–protein interaction (PPI) analysis was employed to identify the principal regulatory proteins under the experimental conditions. The relative quantitative values of each protein in the comparison group samples were subjected to a *t*-test, and the corresponding *p*-values were calculated. Proteins with a corrected *p*-value of <0.05 and a fold change > 1.5 or <1/1.5 were classified as significantly differentially expressed.

### 2.6. Joint Analysis of Transcriptome and Proteome Data

Transcriptomic and proteomic data were subjected to correlation analysis. Nine-quadrant plots were constructed based on the log_2_FC of DEGs and the fold change of DEPs, and their correlation coefficients were calculated using R 4.3.2 software. The significantly correlated DEG and DEP data were mapped to reference pathways in the KEGG database.

## 3. Results

### 3.1. Effects of Interfering with the THRSP Gene on Lipid Formation in C3H10 Cells

**Figure 1:**
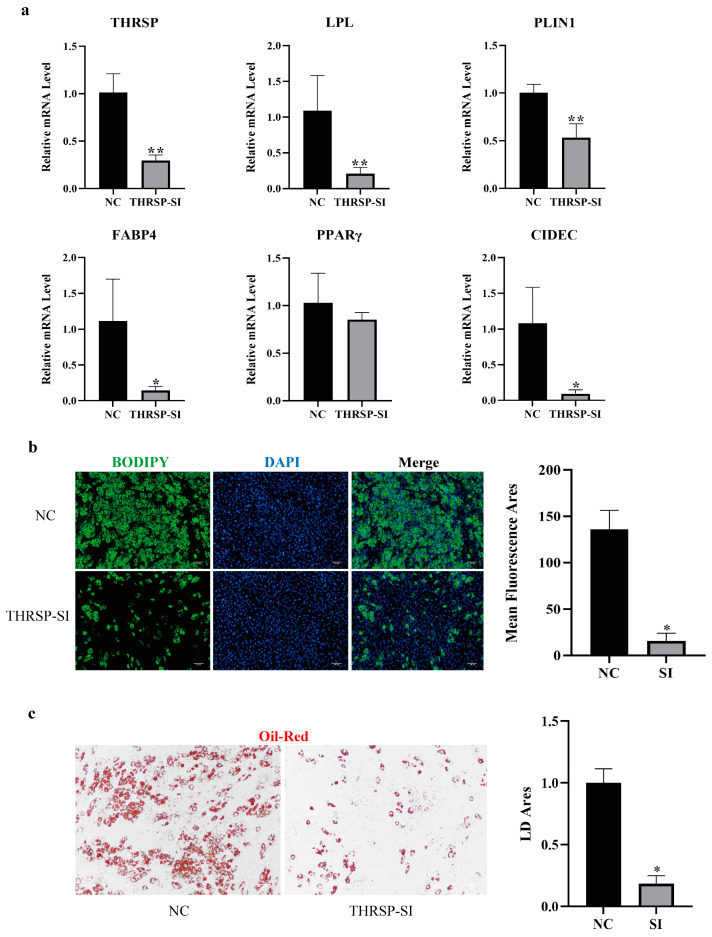
(**a**) Influence of the *THRSP* gene on the adipogenic process; (**b**,**c**) changes in lipid droplet production after interference with *THRSP*. The green and red colors indicate the presence of lipid droplets, while the blue color indicates the presence of nuclei. Bar graphs indicate the average fluorescence area and lipid droplet area, respectively. ** *p* < 0.01, * *p* < 0.05, NC: negative control group, SI: *THRSP* gene silencing group.

We interfered with *THRSP* gene expression in C3H10 cells and found that the expression of lipoprotein lipase (*LPL*), lipid droplet encapsulating protein (*PLIN1*), fatty acid binding protein 4 (*FABP4*), and the cell death-inducing DFF45-like effector C (*CIDEC*, also known as adipose-specific protein 27 (*FSP27*)) were all significantly decreased. The expression of one of the key regulators of adipogenesis (*PPARγ*) was reduced (Figure 1a), but not significantly. The results of lipid droplet staining showed that lipid droplet production was significantly reduced after interfering with the expression of *THRSP* gene (Figure 1b,c). This may indicate that the *THRSP* gene plays a significant role in the regulation of adipogenesis. Therefore, we performed transcriptome and proteome sequencing after perturbation of *THRSP* gene expression to further investigate the specific mechanism by which *THRSP* affects adipogenesis.

### 3.2. Transcriptomics of Interference with THRSP Gene Expression in C3H10 Cells

#### 3.2.1. Quality Control of Sequencing Data, Library Quality Assessment, and Quantitative Gene Expression Analysis

Sequencing error rate checking and GC content distribution checking were mandatory steps after the raw data had been filtered, followed by subsequent analysis of the clean reads obtained. The data are summarized as shown in Table 1. Raw reads, clean reads, clean GC, clean Q20, and clean Q30 content were higher than 40 M, 98%, 96%, and 50%, respectively. The clean reads were quickly and accurately compared to the reference genome, and the number of reads and their percentages compared to the genome exceeded 90% (Table 2), indicating that the reference genome was selected appropriately. The library quality assessment showed that the distribution of sample mapped reads over mRNA transcripts showed a homogenized distribution. The heatmap and PCA results of the correlation of FPKM expression between samples are shown in Figure S1a,b, with high dispersion of samples between groups but high aggregation of samples within groups. After passing the quality control, the next step of the study could be carried out.

#### 3.2.2. Enrichment Analysis ofDEGs

**Figure 2:**
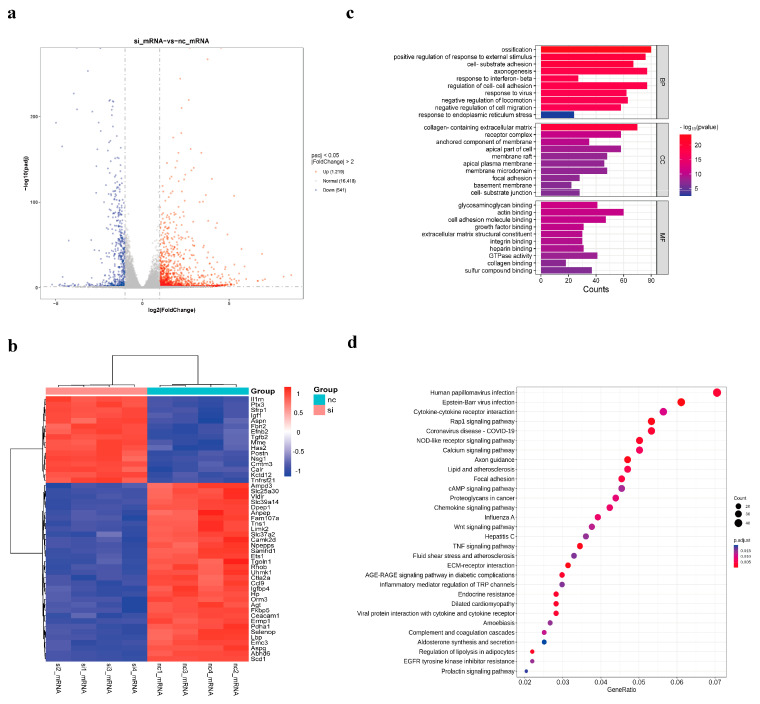
DEGs enrichment analysis by GO and KEGG. (**a**) Volcano plot of DEGs in si and nc groups. Each dot represents a gene. Blue dots are downregulated, red are upregulated, and grey are non-differentially expressed; (**b**) Heatmap of the clustering of the first 50 DEGs in the si and nc groups. Columns represent samples and rows represent genes. Colors represent the expression levels of the genes in the samples; the redder the color, the higher the expression, and the bluer the color, the lower the expression; (**c**) GO analysis of DEGs. The vertical axis is the GO term, the horizontal axis is the number of genes, and the color shows the significance level of the enrichment; (**d**) KEGG enrichment analysis of DEGs. The horizontal axis is the ratio of KEGG-annotated genes to total genes. The bubble size shows the number of genes in the KEGG pathway, and the color shows how significant the enrichment is.

The volcano plot of DEGs is shown in Figure 2a. The heatmaps of DEGs are presented in Figure S1d and illustrate the expression of all upregulated and downregulated genes across all conditions. After interfering with the *THRSP* gene in C3H10, there were a total of 1760 DEGs in the treated and control groups, of which 1219 were upregulated genes and 541 were downregulated genes (Figure S1c). The heatmap shows the top 50 gene expression patterns with the most significant differences in the si vs. nc group (Figure 2b).

GO and KEGG functional enrichment analyses of the differential genes showed that after interfering with the mouse *THRSP* gene, the differentially expressed genes were enriched in biological processes such as ossification, regulation of intercellular adhesion, negative regulation of cell migration, response to ER stress, and interferon β-responses. In terms of cellular composition, they were enriched in the collagen-containing extracellular matrix, receptor complexes, and adhesion plaques, as well as in molecular functions such as actin binding, cell adhesion molecule binding, glycosaminoglycan binding, and GTPase activity. Differential genes in the si vs. nc group were found to be enriched in signaling pathways such as the NOD-like receptor signaling pathway, the calcium signaling pathway, lipids and atherosclerosis, Wnt signaling pathway, cAMP signaling pathway, AGE-RAGE signaling pathway in diabetic complications, and regulation of lipolysis in adipocytes in the KEGG enrichment results. Notably, differential genes have also been associated with diseases such as human papillomavirus infection, Epstein–Barr virus infection, influenza A, and hepatitis C (Figure 2c,d). The identification of transcription factors in differentially expressed genes (DEGs) can facilitate the excavation of differentially expressed transcription factors in different subgroups and their differential regulation mechanisms. *ATF6*, which is related to the ER stress pathway, was a significantly downregulated transcription factor in the si vs. nc group (Table S2). Additionally, the lipid and atherosclerosis signaling pathways, which were markedly enriched in the KEGG, are highly associated with the ER stress pathway.

### 3.3. Astral DIA Quantitative Proteomics Differential Analysis

#### 3.3.1. Enrichment Analysis of DEPs

There were 11,620,000,000 validated spectra, 70,060 peptides, and 7121 specific peptides corresponding to the number of proteins (Table 3). The errors between the actual detection quality and the theoretical quality were generally normally distributed. The peptide length distribution identified by mass spectrometry met the quality control requirements. All samples were analyzed by mass spectrometry with the same parameters, and the overall distribution of signal intensity was basically the same. The amino acid frequency distribution of the identified peptides was consistent with that of the database, and the frequency distribution of arginine R, lysine K, and cysteine C met the quality control requirements (the differences between the frequencies of K and R in the sample and in the database were less than 30%, and the difference between the frequency of C was less than 10%) (Figure S2a–d). The visual heatmap of the Pearson correlation coefficients between two by two of the samples and the schematic results of the quantitative principal component analysis of the proteins were found to be consistent with the RNA-seq results, indicating sample availability (Figure S2e,f).

#### 3.3.2. Identification of DEPs

**Figure 3:**
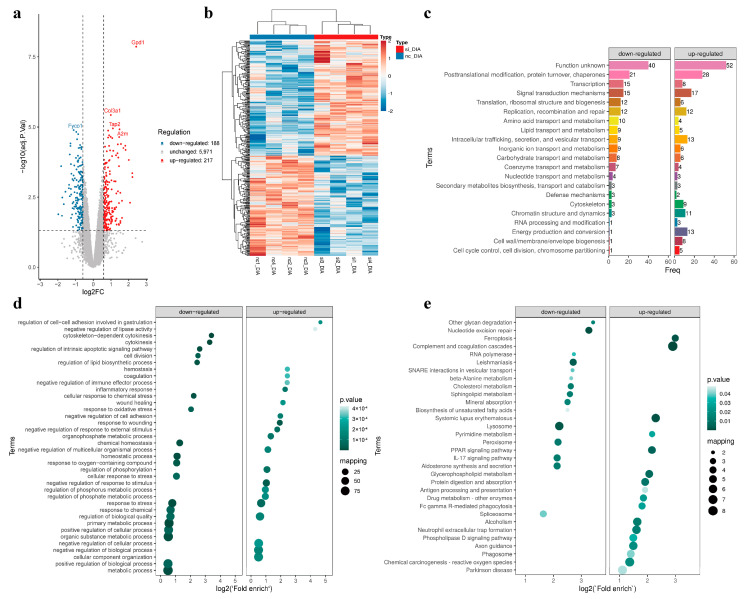
The identification and functional enrichment analysis of DEPs in the si vs. nc groups is presented herewith. (**a**,**b**) DEPs volcano plot and heat map; (**c**) KOG functional classification statistics of DEPs; (**d**) GO enrichment analysis of DEPs; (**e**) KEGG pathway enrichment analysis of DEPs. The size of the bubbles represents the number of members mapped to the KEGG pathway, and the color of the bubbles represents the *p* value.

A total of 6376 DEPs were identified in si vs. nc, with 217 exhibiting upregulated and 188 were demonstrating downregulated (Figure 3a,b). The DEPs were subjected to KOG (clusters of orthologous groups for eukaryotic complete genomes) functional classification statistics, which revealed that the upregulated and downregulated proteins were associated with functions such as translation, signal transduction, lipid transport, and metabolism (Figure 3c). The same protein may assume different functional roles at different subcellular locations. The subcellular localization of a protein can affect its activity, the targets of its interactions, and, consequently, its role within the cell. Subcellular localization analysis revealed that DEPs are localized to the cytoplasm, plasma membrane, cytoskeleton, and organelles (Figure S3a).

Next, we performed GOBP enrichment analysis on DEPs, downregulated DEPs are mainly enriched in the negative regulation of lipase activity, cytoplasmic cleavage, regulation of lipid biosynthesis, and oxidative stress, while upregulated DEPs are mainly enriched in inflammatory response and phosphorus metabolism (Figure 3d). Upregulated DEPs are mainly enriched in MHC class I peptide-loaded complexes, protofibrillar collagen triplexes, and other collagen-associated cellular components. In molecular function, downregulated DEPs are mainly associated with macrolide binding, glycolipid binding, protein–lipid complex binding, lipoprotein particle binding and lipid transfer activity (Figure S3b,c).

The KEGG enrichment results indicated that DEPs were mainly enriched in various signaling pathways, including complement and coagulation cascades, leishmaniasis, β-alanine metabolism, cholesterol metabolism, sphingolipid metabolism, peroxisomes, the PPAR signaling pathway, and glycerophospholipid metabolism (Figure 3e). A relatively independent spatial structural domain exists in mature protein molecules; therefore, the analysis of structural domains can predict the function of proteins, protein–protein interactions, and drug action target regions. The results showed that DEPs were enriched in the C-terminal structural domain of protofibrillar collagen, the structural domain of phosphatidylinositol, the macroglobulin domain MG3, the structural domain of calcium-binding EGF, the structural domains of the protein complexes, and the serine and threonine kinases (Figure S3d). Interestingly, the structural domains associated with megaglomerin were significantly enriched in upregulated DEPs. The best-studied macroglobulin is immunoglobulin M (IgM), which is an aggregation of five immunoglobulin monomers. Although immunoglobulin M cannot pass through the placenta, it is very efficient at agglutinating foreign bodies and binding complement, and it represents the initial line of defence against the intrusion of foreign bodies. Thesecond is α2-macroglobulin, a protease inhibitor. It is worth noting that there are many other lipoproteins that are also macroglobulins. These results suggest that the *THRSP* gene may be significantly associated with a number of biological processes, including fibrillar collagen formation, lipid anabolism, and cholesterol metabolism.

DEP interactions were obtained by comparison with the STRING (v.11.0) protein interaction network database (Figure S4). Among them, SCD1 and DGAT2 are known to be associated with the regulation of the lipid droplet genesis process.

### 3.4. Joint Analysis of Transcriptomic and Proteomic Data

**Figure 4:**
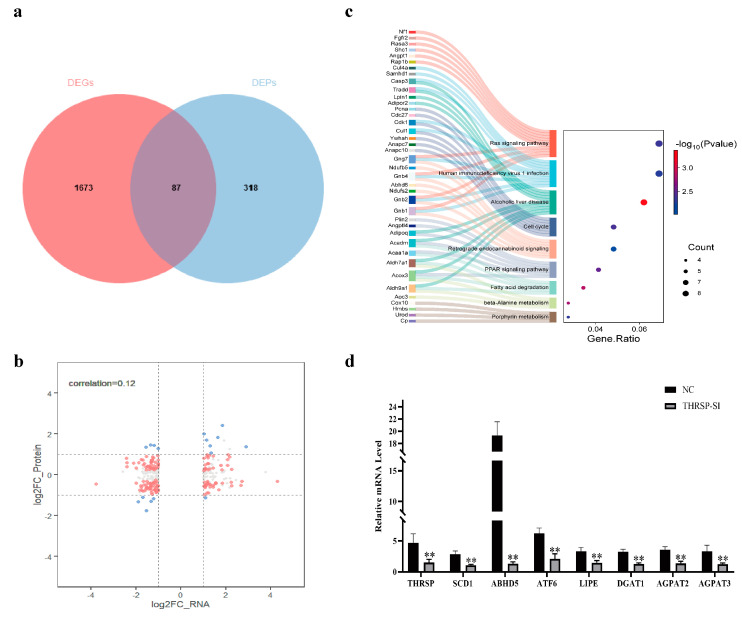
Correlation analysis of transcriptomics and proteomics. (**a**,**b**) Venn and nine-quadrant plots of DEGs versus DEPs. (The horizontal coordinate is the differential fold of the transcriptome and the vertical coordinate is the differential fold of the proteome. The horizontal/vertical coordinate dashed lines indicate the differential fold thresholds of the transcriptome/proteome. Each dot represents a gene/protein, with gray dots indicating non-differentiated proteins and genes, blue dots indicating genes and proteins that are both significantly different (up-regulated or down-regulated), and red dots indicating genes/proteins for which one histology is significantly different and one is not); (**c**) Sankey plot showing significant correlation of DEGs with DEPs enriched to the top ten KEGG pathways. The Sankey plot on the left side of the figure shows the genes and their enriched pathways. The bubble plot on the right side depicts the number of genes, with bubble size indicating the relative abundance of each gene. The color shade represents the *p* value; (**d**) Validate the expression of differentially expressed genes and key genes through RT-qPCR. ** *p* < 0.01.

**Figure 5:**
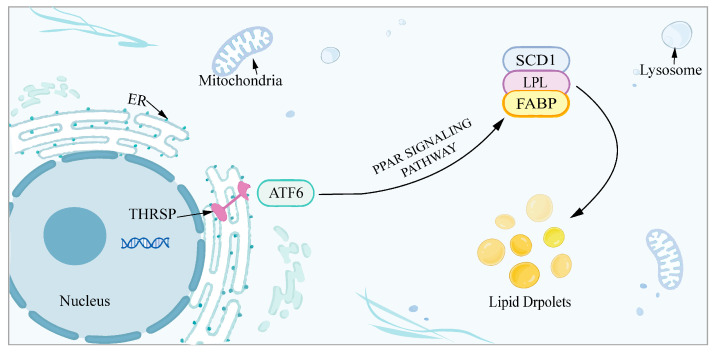
The potential mechanism by which *THRSP* affects lipogenesis.

Analysis of the differential genes obtained from the transcriptome and proteome revealed that 87 out of 318 DEPs matched DEGs, including 50 downregulated DEPs, 37 upregulated DEPs, and 80 DEPs that had the same expression profile as DEGs (Figure 4a). Correlation analysis of DEGs and DEPs (Figure 4b) showed a correlation coefficient of 0.12, which may be due to the presence of translational regulation. KEGG pathway analysis of related DEPs and DEGs revealed significant enrichment for alcoholic liver disease, fatty acid degradation, β-alanine metabolism, the PPAR signaling pathway, the cell cycle, the ras signaling pathway, porphyrin metabolism, human immunodeficiency virus 1 infection, retrograde endocannabinoid signaling, and arginine and proline metabolism (Figure 4c). These are associated with adipogenesis and lipid metabolism. The expression levels of seven differential genes were quantified using RT-qPCR. The results showed that *SCD1*, *ABHD5*, *ATF6*, *LIPE*, *DGAT1*, *AGPAT2*, and *AGPAT3* were all significantly downregulated in the si group (Figure 4d). Previous studies have demonstrated a significant downregulation of *FABP4* expression following the interference of *THRSP*. Both *FABP4* and *SCD1* have been identified as key factors within the PPAR signaling pathway, while the transcription factor *ATF6* has also been shown to undergo significant downregulation. Based on these observations, it was suggested that *THRSP* may be associated with ER stress and the PPAR signaling pathway (Figure 5).

## 4. Discussion

The relationship between metabolic diseases, diabetes mellitus, NAFLD, lipids, and atherosclerosis is a complex and interrelated field involving multiple aspects of fat deposition, lipid metabolism, and ER stress [32,33,34,35,36]. *THRSP* is involved in lipid metabolic processes through interactions with key lipid synthases and proteins. The use of thyroid hormone receptor β (THR-β) agonists has been demonstrated to be an effective treatment for dyslipidemia and NAFLD; reductions in thyroid hormone may exacerbate both conditions [37,38]. Thus, *THRSP*, the thyroid hormone-responsive protein, is closely associated with metabolic disorders resulting from lipid metabolism. In addition, *THRSP* is indispensable for fat synthesis in mammalian mammary glands. *THRSP* expression is upregulated in the mammary gland of mice during lactation, whereas deletion of *THRSP* decreases mammary fat synthesis capacity [39]. Particularly, *THRSP* polymorphisms have been associated with mammalian, avian, and rodent adipogenesis, and as a key candidate gene for fat deposition in different pig breeds, *THRSP* has been identified in several histologies [40,41,42]. In this study, differential RNA-seq analysis of C3H10 cells interfering with *THRSP* showed altered *THRSP* gene expression involved in the calcium signaling pathway, lipids and atherosclerosis, ER stress, the cyclic phosphatase signaling pathway, the AGE-SYS signaling pathway in diabetic complications, the Wnt signaling pathway, and the regulation of lipolysis in adipocytes. Disease pathways such as influenza A and hepatitis C are also included. Atherosclerosis and diabetes mellitus are typical metabolic diseases, and metabolic diseases still have a high global prevalence [43,44,45]. Lipid deposition and metabolic abnormalities are inextricably linked to them [46,47]. Therefore, studying the function of *THRSP* in lipid metabolism is particularly important in ameliorating lipid-related metabolic diseases.

In this study, differential genes were significantly enriched in the lipid metabolic disease class, and *ATF6*, the key factor of ER stress, was significantly downregulated after *THRSP* interference. This suggests that *THRSP* is associated with the interplay between ER stress and lipid metabolism. The ER plays a significant role in the synthesis and processing of lipids, including phospholipids, triglycerides, and cholesterol. ER stress activates multiple signaling pathways (e.g., *IRE1*, *PERK*, and *ATF6*) [48,49]. These pathways can regulate the expression of lipid metabolism-related genes through different mechanisms, leading to changes in intracellular lipid metabolism [5,50,51,52]. ER stress induces a response that promotes lipid synthesis and storage while inhibiting lipid catabolism and excretion. This response leads to further deposition of fat in the liver, aggravating the condition of NAFLD [53]. Reducing the expression of *THRSP* may have a mitigating effect.

The proteomic results revealed that DEPs were significantly enriched for processes such as lipid biosynthesis regulation, phosphorus metabolism, glycolipid binding, lipoprotein particle binding, protein–lipid complex binding, lipid transfer, cholesterol metabolism, sphingolipid metabolism, and glycerophospholipid metabolism, as well as for the PPAR signaling pathway. Among them, PPAR is a group of nuclear receptors involved in the regulation of lipid metabolism and energy homeostasis [54,55]. *THRSP* may affect adipogenesis by influencing the expression or activity of PPAR. *PPARγ* is a key regulator of adipocyte differentiation and lipid storage [56]. *THRSP* promotes adipogenesis by affecting downstream target genes, such as *PPARγ* and other genes related to fatty acid synthesis and adipocyte differentiation. By regulating the expression of genes involved in lipid metabolism, *THRSP* can help cells adapt to the ER stress environment, thus maintaining cellular energy homeostasis in stressful situations. ER stress is closely related to adipogenesis, and sustained ER stress interferes with the unfolded protein response (UPR), which in turn affects adipocyte differentiation and lipid accumulation. The PPAR signaling pathway may play a key role in metabolic diseases, such as diabetes and obesity, through its regulation of the interconnection between ER stress and adipogenesis [12,57].

The results of the combined transcriptome and proteome analysis in this study showed that significantly associated DEGs and DEPs were enriched in the pathways of alcoholic liver disease, β-alanine metabolism, porphyrin metabolism, the PPAR signaling pathway, the ras signaling pathway, fatty acid degradation, human immunodeficiency virus 1 infection, retrograde endogenous cannabinoid signaling, and arginine and proline metabolism, and that after interfering with *THRSP* expression. Altered ER stress states can trigger pathways associated with inflammation. Among the results of transcriptomic DEGs after interference with *THRSP*, differential genes were selected for validation. The results showed significant downregulation of the following genes associated with lipid droplets: *SCD1*, *ABHD5*, *ATF6*, *LIPE*, *DGAT1*, *AGPAT2*, and *AGPAT3* [58,59,60,61]. In addition, in previous studies, lipid synthesis-related genes, such as *LPL*, *FABP4*, *PLIN1* and *CIDEC*, were significantly downregulated and lipid droplet production was markedly reduced after interfering with *THRSP* in C3H10 cells. Combined with the above analysis, the results demonstrate that *THRSP* is closely related to lipid metabolism. It is noteworthy that, although we investigated the effects of interfering with *THRSP* on the expression of key genes and the changes in cellular phenotype, and explored the mechanism of this process through the transcriptome and proteome, the experimental evidence for this mechanism remains to be explored and investigated. It is our contention that this will provide new theoretical support for understanding the mechanism by which *THRSP* affects lipid synthesis and metabolism. Furthermore, this will assist in the treatment of lipid metabolism-related diseases.

## 5. Conclusions

We showed that *THRSP* may affect lipid synthesis and metabolism by regulating the ER stress factor *ATF6* and subsequently through the PPAR signaling pathway. By interfering with the expression of the *THRSP* gene, the occurrence of abnormal lipid metabolism diseases associated with ER stress can be ameliorated. However, the key genes and mechanisms specifically affected by *THRSP* still need to be investigated further.

## Figures and Tables

**Table 1:** Sequencing data filtering and statistics.

Sample	Raw Reads	Clean Reads	Clean GC (%)	Clean Q20 (%)	Clean Q30 (%)
nc1_mRNA	41,493,716	41,168,042	51.05	98.76	96.14
nc2_mRNA	45,440,574	45,096,558	51.38	98.79	96.23
nc3_mRNA	42,608,278	42,274,140	51.51	98.83	96.37
nc4_mRNA	41,629,818	41,340,084	51.81	98.86	96.45
si1_mRNA	40,936,016	40,639,934	51.02	98.8	96.25
si2_mRNA	42,610,358	42,267,558	50.05	98.78	96.21
si3_mRNA	42,220,118	41,895,614	50.72	98.78	96.21
si4_mRNA	40,863,570	40,517,122	50.4	98.74	96.1

**Table 2:** Statistical table of sample–reference genome comparisons.

Sample	Total_Reads	Total_Map	Unique_Map	Multiple_Map
si1_mRNA	40,639,934	39,277,420 (96.65%)	37,146,966 (91.41%)	2,130,454 (5.24%)
si2_mRNA	42,267,558	40,737,280 (96.38%)	38,181,464 (90.33%)	2,555,816 (6.05%)
si3_mRNA	41,895,614	40,360,230 (96.34%)	38,048,698 (90.82%)	2,311,532 (5.52%)
si4_mRNA	40,517,122	38,917,762 (96.05%)	36,607,570 (90.35%)	2,310,192 (5.7%)
nc1_mRNA	41,168,042	39,709,460 (96.46%)	37,510,576 (91.12%)	2,198,884 (5.34%)
nc2_mRNA	45,096,558	43,526,588 (96.52%)	41,189,602 (91.34%)	2,336,986 (5.18%)
nc3_mRNA	42,274,140	40,741,620 (96.37%)	38,454,064 (90.96%)	2,287,556 (5.41%)
nc4_mRNA	41,340,084	40,041,226 (96.86%)	38,146,818 (92.28%)	1,894,408 (4.58%)

**Table 3:** Statistics of Proteomics Analysis.

MS/MS	MS/MS Identified	Peptide Identified	MS/MS Identified [%]	Identified Protein
242,209,476,608	11,620,000,000	70,060	4.8	7121

## Data Availability

Raw data can be obtained by contacting the corresponding author.

## Supplementary Materials

The following supporting information can be downloaded at: https://www.mdpi.com/article/10.3390/genes15121562/s1, Figure S1: Correlation and differential gene statistics of samples in transcriptome data; Figure S2: Quality control analysis of proteomic sequencing data; Figure S3: Functional analysis of DEPs; Figure S4: STRING (v.11.0) protein interaction network diagram showing DEPs interacting with each other. Table S1: Primes list; Table S2: Differential gene transcription factor annotation results.
