# Supplementary material for: Integrative Transcriptomics and Proteomics Analysis Reveals THRSP’s Role in Lipid Metabolism
Source: Genes (Basel). 2024 Nov 30;15(12):1562. doi: 10.3390/genes15121562 (PMC11675175; doi:10.3390/genes15121562)
Supplement: Supplementary file 1 [file genes-15-01562-s001.zip › Figure S.pdf]

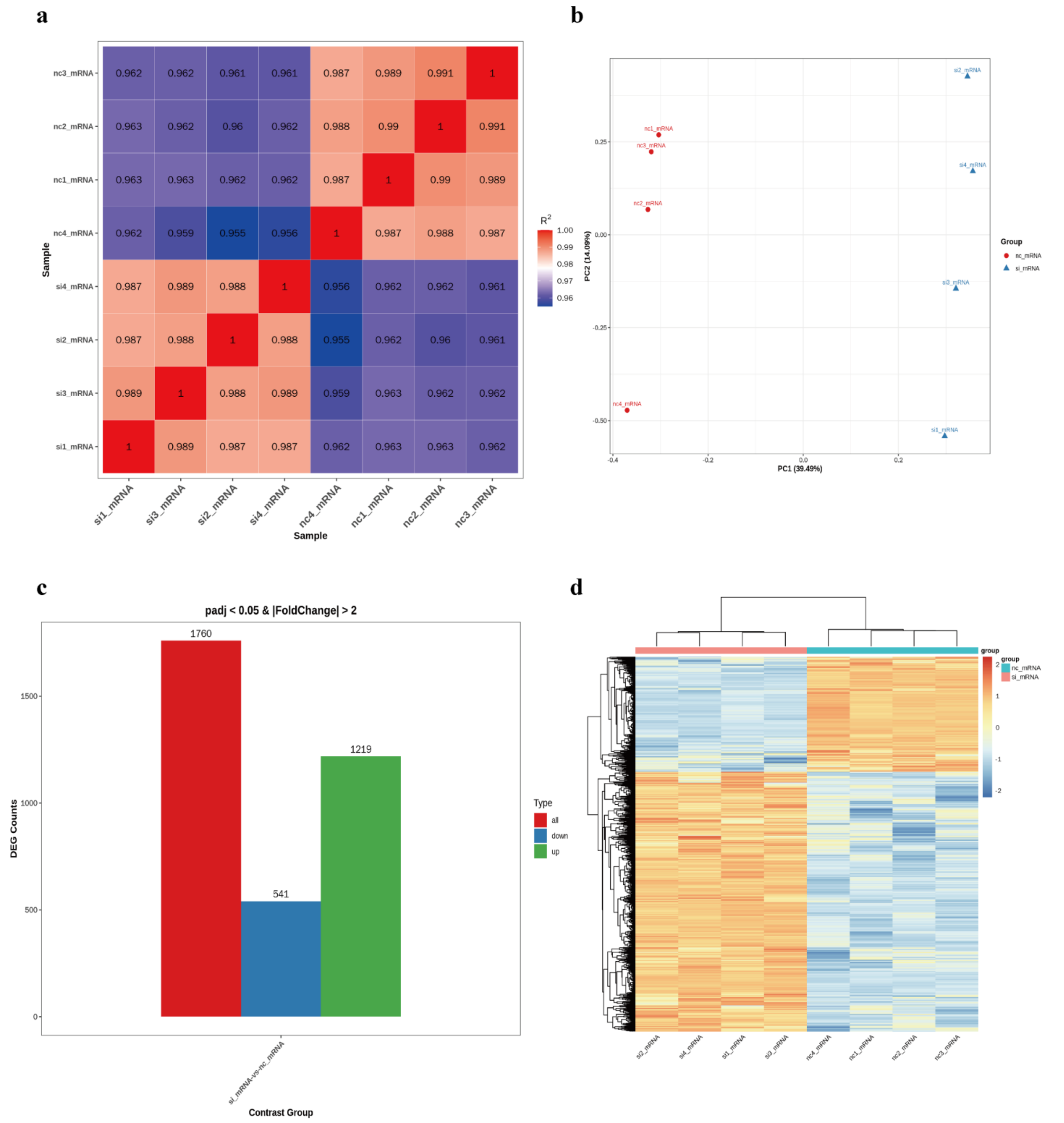

Figure S1. Correlation and differential gene statistics of samples in transcriptome data. (a) Heat map of correlation of FPKM expression between samples. The value on each color block represents the square of the Pearson's correlation coefficient between two samples on the corresponding horizontal and vertical axes of the color block, and the larger the value, the higher the correlation. (b) Plot of the results of principal component analysis of FPKM expression. Percentages indicate the contribution value of the corresponding principal components to the sample differences, each point represents one sample, and different groupings of samples are represented by different colors and shapes. (c) Histogram of the statistics of the number of DEGs in each comparison combination. Red is all DEGs, green is up-regulated genes, and blue is down-regulated genes. (d) Heatmap of differential expression clustering. Different columns in the graph represent different samples and different rows represent different genes. The colors represent the expression levels of the genes in the samples; the redder the color, the higher the expression, and the bluer, the lower the expression.

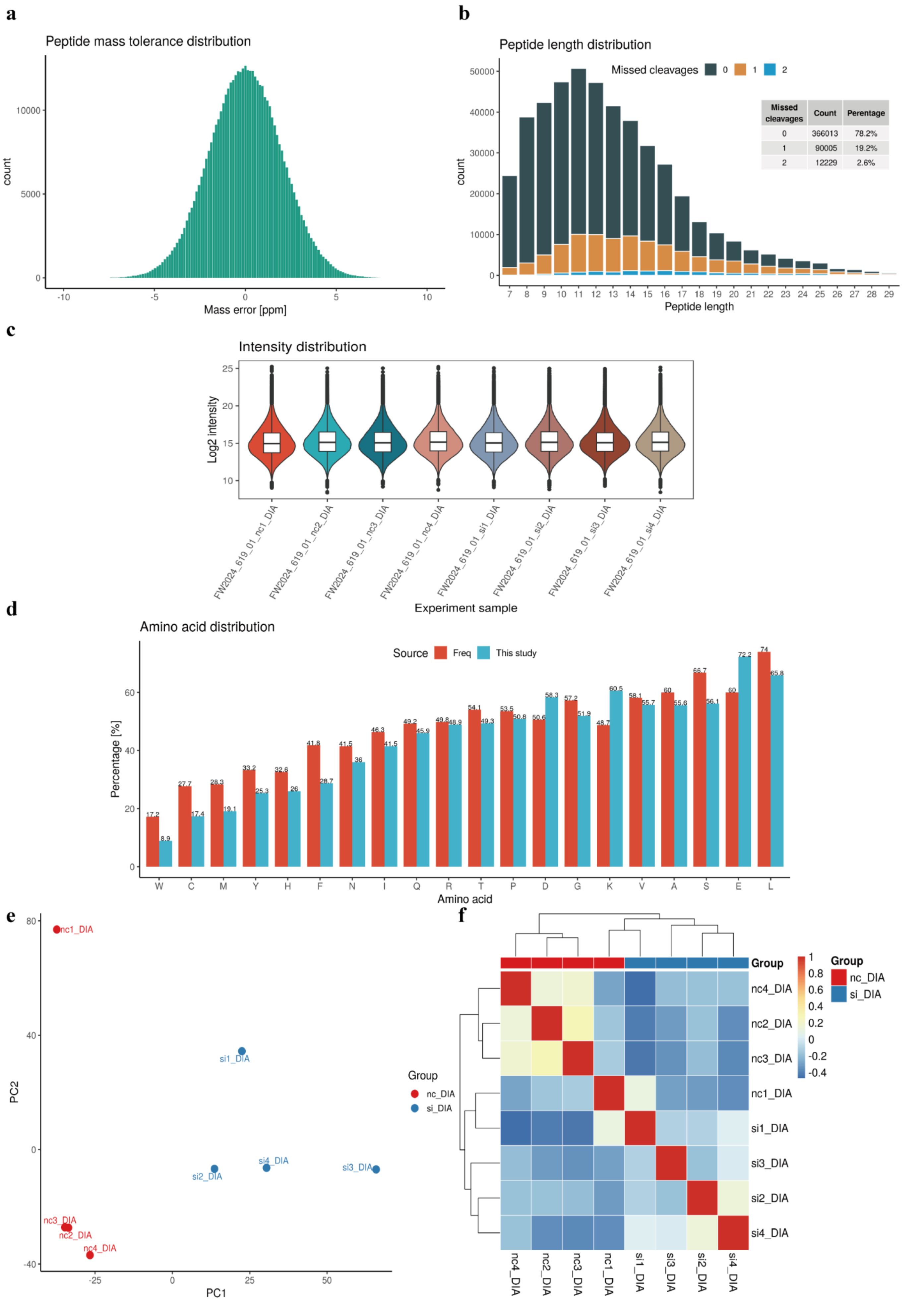

Figure S2. Quality control analysis of proteomic sequencing data. (a), (b), (c) and (d) indicate the peptide mass error distribution,

peptide length distribution, peptide signal intensity distribution and peptide amino acid frequency results, respectively. (e) and (f) Sample repeatability test. Repeatability was assessed by the statistical analysis methods of PCA and PCC between all samples two by two, respectively.

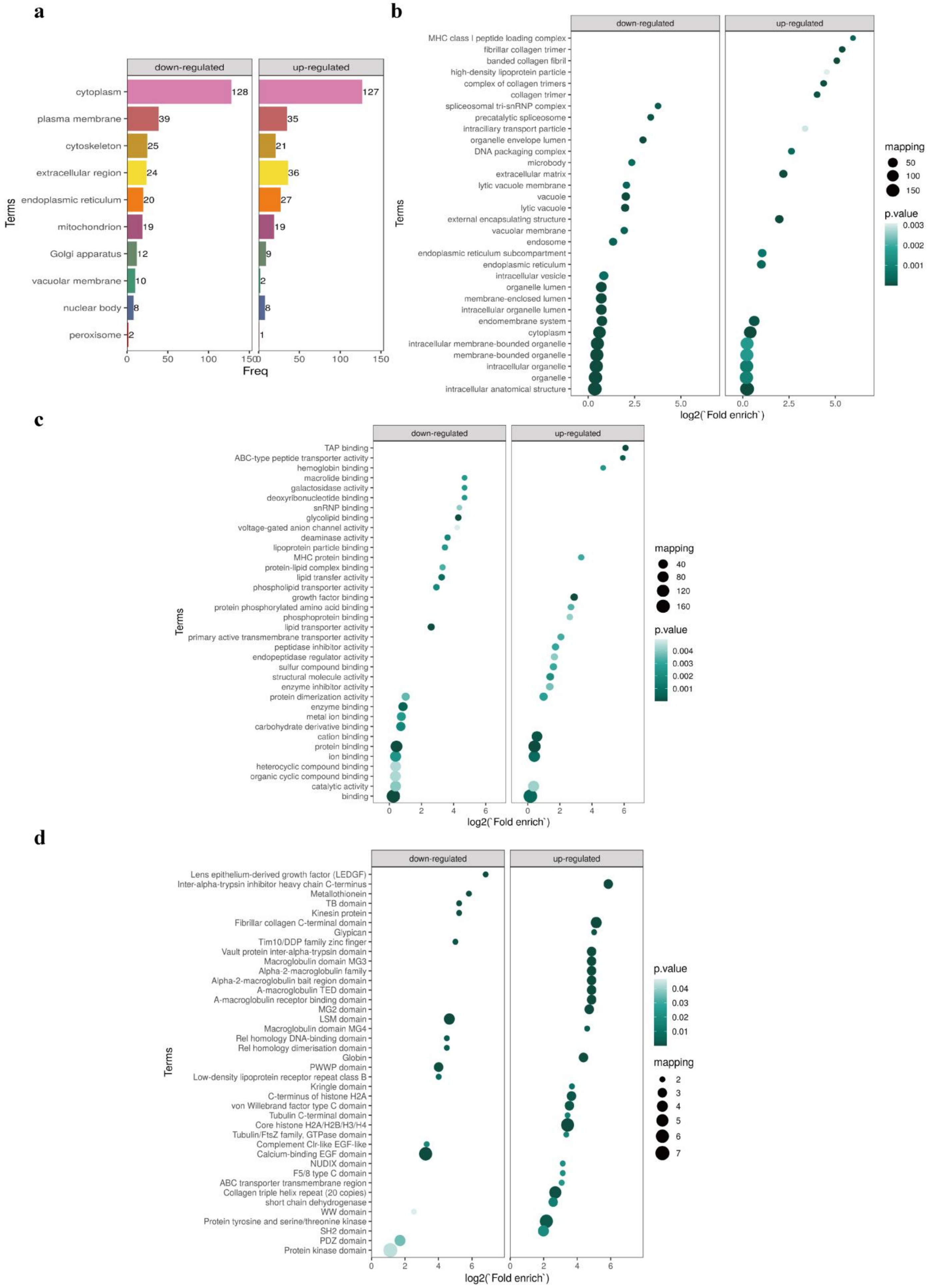

Figure S3. Functional analysis of DEPs. (a) Subcellular structure annotation. (b) and (c) Cellular component and Molecular function enrichment analysis in GO terms of DEPs. (d) Protein structural domain enrichment bubble diagram of DEPs.

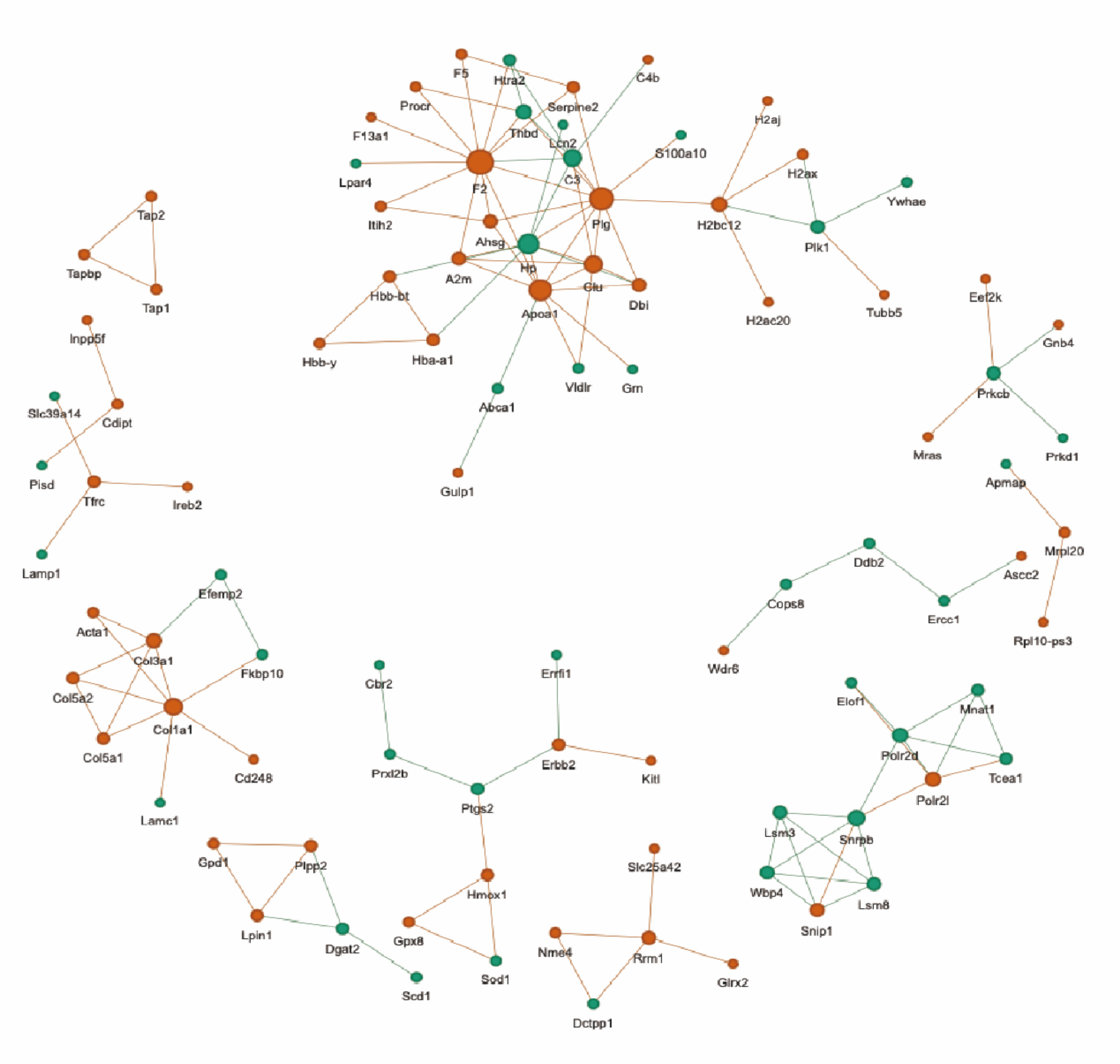

Figure S4. STRING (v.11.0) protein interaction network diagram showing DEPs interacting with each other.
